# Diagnostic usefulness of 10-step tandem gait test for the patient with degenerative cervical myelopathy

**DOI:** 10.1038/s41598-021-96725-6

**Published:** 2021-08-26

**Authors:** Dallah Yoo, Kyung-Chung Kang, Jung-Hee Lee, Ki Young Lee, In-Uk Hwang

**Affiliations:** 1 Department of Orthopaedic Surgery, Kyung Hee University Hospital, College of Medicine, Kyung Hee University, 23 Kyungheedaero, Dongdaemun-gu, Seoul, 02447 Republic of Korea; 2 Department of Neurology, Kyung Hee University Hospital, College of Medicine, Kyung Hee University, Seoul, Republic of Korea

**Keywords:** Signs and symptoms, Neurological manifestations, Spinal cord diseases

## Abstract

Tandem gait is considered one of the most useful screening tools for gait impairment. The aim of this study is to evaluate diagnostic usefulness of 10-step tandem gait test for the patients with degenerative cervical myelopathy (DCM). Sixty-two DCM patients were compared to 55 persons without gait abnormalities as control. We counted the number of consecutive steps and graded into five according the number of steps and stability. Five grades of tandem gait were investigated for association with clinical parameters including qualitative Japanese orthopedic association (JOA) sub-score for lower extremities and Nurick scale and quantitative balance and gait assessments. The number of tandem steps were reduced and the grades of tandem gait were differently distributed in the DCM patients compared to controls (steps, 7.1 ± 3.6 versus 9.9 ± 0.4, *p* < 0.001; grades of 0/1/2/3/4/5, 1/13/14/15/19 versus 0/0/2/15/38, *p* < 0.001 in patients with DCM and control respectively). Patients with DCM showed more unstable balance and abnormal gait features including slower velocity, shorter strides, wider bases with increased stance phase of a gait cycle compared to the control group. The grades of tandem gait were correlated with JOA sub-score (r = 0.553, p < 0.001) and the Nurick scale (r = − 0.652, p < 0.001) as well as both balance and gait parameters. In DCM patients, tandem gait was impaired and correlated with severity of gait abnormality. The authors believe that 10-step tandem gait test is an objective and useful screening test for evaluating gait disturbance in patients with DCM.

## Introduction

Gait disturbance is one of the cardinal symptoms of degenerative cervical myelopathy (DCM)^1–3^. For the assessment of gait abnormality in patients with DCM, the Japanese Orthopedic Association (JOA) Score and Nurick scale have been used around the world^4–7^. However, sometimes it is challenging to accurately distinguish the grades of these scores because they are based on subjective questionnaires and elusive differences between the grades.

Recently, the use of gait analysis for evaluating lower extremity kinematics and gait impairment has increased^8^. Some studies have been conducted on gait dysfunction in patients with cervical myelopathy. Kalsi-Ryan et al.^1^ showed promising results of quantitative gait assessments as an accurate and objective method to diagnose and classify DCM. Haddas et al.^9^ demonstrated a relationship between aberrant spinal alignment and lower extremity function in cervical myelopathic patients. However, the clinical utilization of gait analysis is low because of cost and space constraints and the lack of established standards.

In practice, tandem gait testing is considered a feasible and useful neurological examination to assess imbalance and gait impairment^10,11^. However, as far as we know, there is no standardized scoring system of tandem gait testing to help quantify imbalance and gait dysfunction of patients with DCM. In this study, we introduced a 10-step tandem gait testing and five grades according to the number of steps and stability and hypothesized that this could be a useful screening test for evaluating the gait impairment of DCM patients.

The aim of this study was to evaluate whether the 10-step tandem gait test and grading can be a useful diagnostic tool to check imbalance and gait impairment of patients with DCM.

## Methods

### Patient selection and study design

From August 2019 to March 2020, patients that consecutively underwent surgery for DCM, including cervical spondylotic myelopathy, ossification of posterior longitudinal ligament (OPLL), and herniated intervertebral disc at our institution were reviewed and the data were prospectively collected. The inclusion criteria were: (1) symptomatic cervical myelopathy (hand clumsiness, gait disturbance, whole body paresthesia and/or bowel and bladder dysfunction); and (2) severe cervical canal narrowing due to disc-osteophyte complexes, ossification of the posterior longitudinal ligament and/or hypertrophy or buckling of the ligamentum flavum in magnetic resonance (MR) and computed tomography (CT) images. Patients with other problems affecting their gait, such as other neurologic abnormalities (Parkinson’s disease, poliomyelitis sequelae, and cerebrovascular disease) or symptomatic joint problems (osteoarthritis of the hip, knee, and ankle) were excluded from this study. To verify gait impairments of the patients, we compared the patients with DCM to subjects without gait abnormality as a control group. The control group was selected as follows: (1) over 20 years of age; (2) individuals who visited orthopedic department during same period with experiment group; (3) patients who complained cervical radiculopathy symptoms without gait impairment; and (4) with all data including demographic, scorings for gait abnormality, and the results of balance test and gait analysis.

### Assessment of gait disturbance

Japanese Orthopedic Association (JOA) sub-score for lower extremities (score range: 0–4) and Nurick scale (grade range: 0–5) were assessed preoperatively. The 10-step tandem gait test was also performed and we defined the five grades of the 10-step tandem gait test as follows: grade 0 (impossible to walk), grade 1 (≤ 3 steps), grade 2 (< 10 steps), grade 3 (10 steps, but unstable with swaying from side to side), and grade 4 (10 steps, normal), and compared with the JOA sub-scores (Table 1).

**Table 1:** Comparisons of each item between JOA sub-score for lower extremity and tandem gait grade.

Score/grade	JOA sub-score (lower extremities)	Tandem gait grade
0	Impossible to walk	Impossible to walk
1	Needs cane or assistance on flat surface	≤ 3 steps
2	Needs assistance on stairs	< 10 steps
3	Walks unaided, but slowly	10 steps, unstable/sway
4	Normal	10 steps, normal

### 10-Step tandem gait test

**Figure 1:**
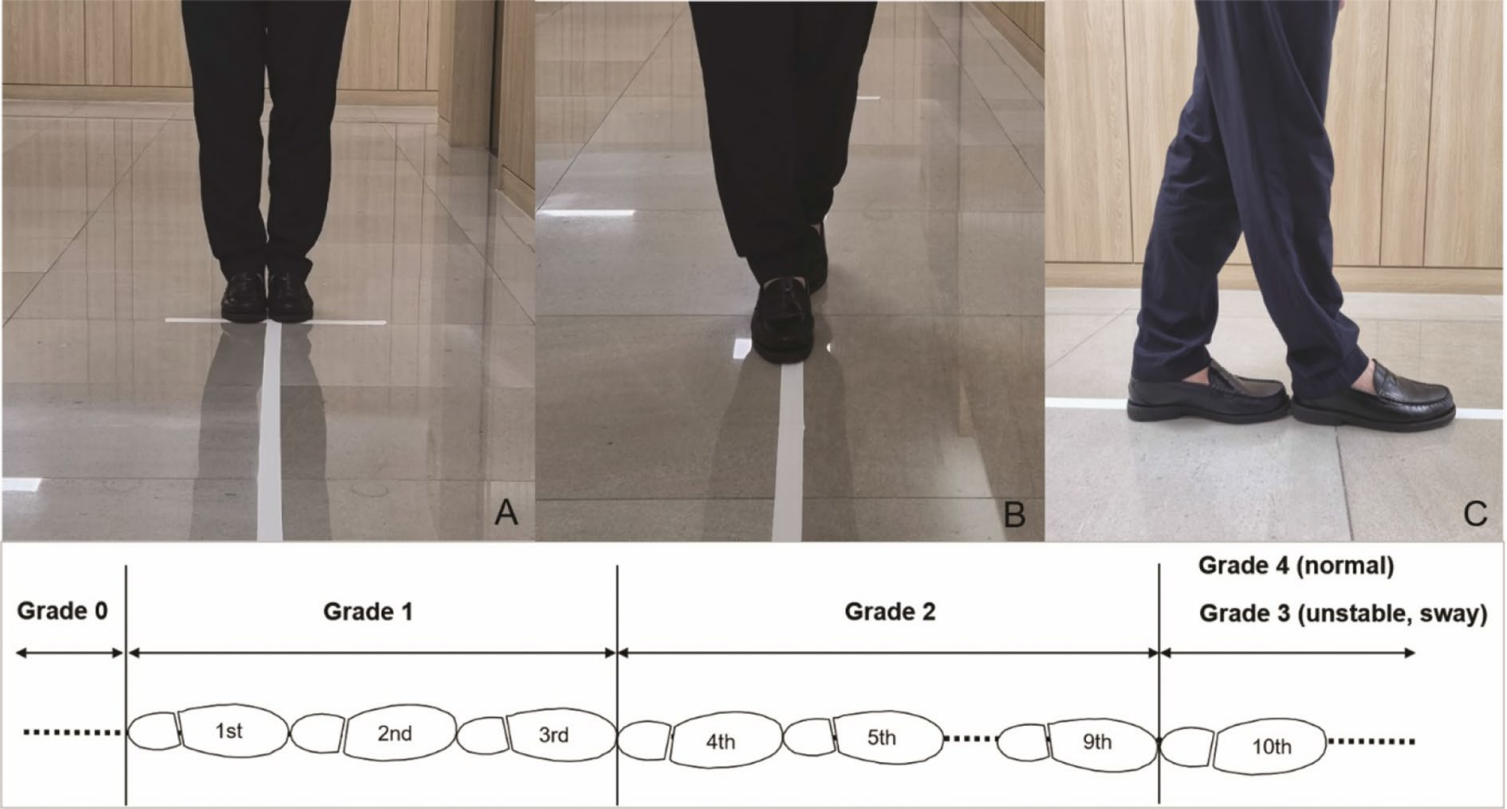
Photographs for a toe-to heel touch of 10-step tandem gait. Before the test, the patients stand comfortably with their feet together (**A**) and carefully walk 10 steps in a straight lined-up flat floor like the photos [(**B**) frontal and (**C**) side]. At the bottom, schematic diagram is the detailed 10-step tandem gait grading. According to the number of consecutive steps of the test, the patients were sub-grouped into five grades: grade 0 (impossible to walk), grade 1 (≤ 3 steps), grade 2 (< 10 steps), grade 3 (10 steps, but unstable with swaying from side to side), and grade 4 (10 steps, normal).

The patients performed 10-step tandem gait tests. Before the tests, the patients stand comfortably with their feet together and carefully walk 10 steps in a straight lined-up flat floor. They made their best effort to make a toe-to-heal touch with each step at their own velocity (Fig. 1). The number of steps made before the first misstep was counted. After first 10-step tandem gait test, the patient turned around and repeated the tandem gait test in the same line and the number of steps was counted in the same manner. Of the two tandem gait tests, the one with a higher tandem gait grade was assessed.

### Gait and balance analyses

All participants performed static and dynamic balance test (NeuroCom BASIC Balance Master, Natus Medical Inc., Pleasanton, CA, USA) and a treadmill-based gait analysis (FDM-T, Zebris Medical GmbH, Isny Im Allgäu, Germany). All participants were asked to walk on the treadmill for 30 s with maximal comfortable treadmill speed (MCTS)^12^, and the system records spatiotemporal parameters including MCTS (cm/s), stride time (s), step/stride length (cm), step width (cm), cadence (steps/min), and the proportion of stance/swing/double stance/singling limb support phase (%) of a gait cycle.

**Figure 2:**
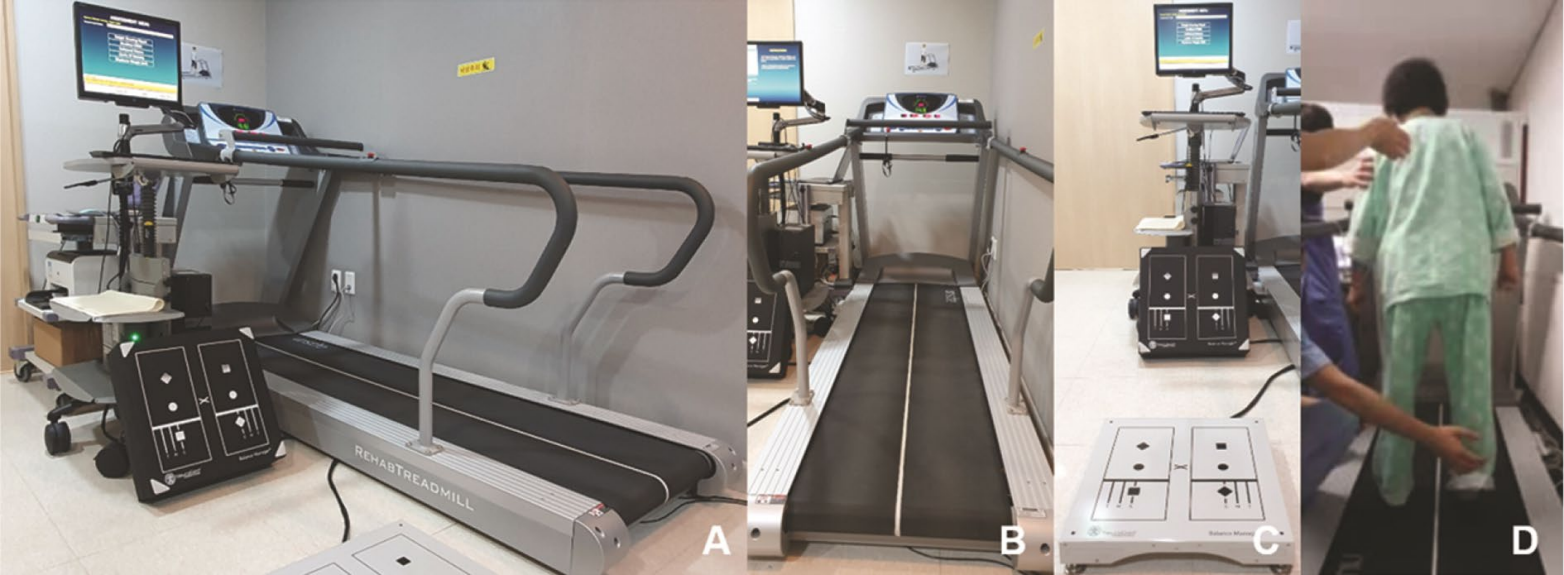
Photographs of instruments for gait and balance analyses. Whole figure of machines and setting of gait analysis room were shown (**A**). Each instrument for gait (**B**) and balance (**C**) analysis lied at the side and in case of unstable gait of the patient, the test was performed with special care to keep the patient from slipping down (**D**).

The balance tests are composed of modified clinical tests of sensory integration and balance (mCTSIB), limits of stability (LOS), and rhythmic weight shift (RWS) (Fig. 2)^13–15^. In the mCTSIB, the participants were administered to stand with their hands at their sides and performed the following four conditions: standing on a firm surface with eyes open, a firm surface with eyes closed, a foam surface with eyes open, and a foam surface with eyes closed. Static stability was measured as the mean center of gravity (COG) sway velocity (deg/s) that the displacement of COG is recorded as degrees for 10 s in each trial and the scores of the three trials were averaged. The mCTSIB was terminated when subjects were unable to maintain their position and the score of failed tests is recorded as the maximal value of 4.0 deg/s.

In the LOS test to assess dynamic stability, the participants stood on a designated foot position about a hip’s width apart on a fixed firm plate, and the position was adjusted so that the displayed COG was positioned within the center square on the computer screen. When the cue sounded, the participants moved to shift their weight to the eight target squares one by one, which were arranged in an elliptical orbit at calculated intervals based on the individual height, as fast and accurately as they could. The test measured the reaction time (time elapsed between command and movement), velocity (deg/s), end-point or maximal excursion of the target (%), and directional control (the mean percentage of COG movement in the right direction) for each of the eight directions. Composite scores for each parameter were generated by averaging the results of the tests performed in each of the eight directions.

The RWS test evaluates the ability to move the COG mediolaterally and anteroposteriorly between two targets (set by 50% of individual LOS) at three different speeds (slow, medium, and fast). The subjects were instructed to shift their weight following the cursor displayed on the screen. The outcome variables were the mean COG movement speed (deg/s) and directional control (%). Composite scores for COG velocity and directional control were generated by averaging all trials performed in two directions.

### Statistical analysis

The baseline characteristics (Table 2) are described as mean, standard deviation, frequency (percentage), and the range. Group comparisons were analyzed by independent *t* test or Mann–Whitney test for continuous variables depending on whether the assumption was satisfied. For categorical variables, chi-squared test was performed for sex and otherwise, Fisher’s exact test was applied. Spearman’s rank correlation analysis was performed to determine associations between the clinical scales and variables of balance and gait analysis. Statistical analyses were performed using SPSS (Version 25.0, SPSS Inc., Chicago, IL, USA) at an alpha level of 0.05.

**Table 2:** Comparisons of clinical features between patients with degenerative cervical myelopathy (DCM) and radiculopathy.

	DCM	Control	*p*-value
Number	62	55	
Age, years	59 ± 12, 27–82	56 ± 11, 24–75	0.103^a^
Sex, M (%)	41 (66.1)	35 (63.6)	0.778
Weight (kg)	67.5 ± 11.2	66.3 ± 8.91	0.821
Height (cm)	163.6 ± 7.9	165.4 ± 9.3	0.230
BMI (kg/m^2^)	25.2 ± 3,2	24.2 ± 2.6	0.113
Tandem gait, steps (0–10)	7.1 ± 3.6, 0–10	9.9 ± 0.4, 8–10	**< 0.001**
Tandem gait, grade (0/1/2/3/4) (%)	1/13/14/15/19 (1.6/21.0/22.6/24.2/30.7)	0/0/2/15/38 (0/0/3.6/27.3/69.1)	**< 0.001**
JOA subscore (lower limb) (0/1/2/3/4) (%)	1/4/16/22/19 (1.6/6.5/25.8/35.5/30.6)	0/0/0/0/55 (0/0/0/0/100)	**< 0.001**
Nurick scale (0/1/2/3/4/5) (%)	8/19/20/8/0/0 (14.5/34.5/36.4/14.5/0/0)	28/25/1/1/0/0 (50.9/45.5/1.8/1.8/0/0)	**< 0.001**
Balance parameters			
mCTSIB			
Firm, eye-opened, deg/s	0.5 ± 0.3	0.3 ± 0.1	**< 0.001**
Firm, eye-closed, deg/s	0.8 ± 0.6	0.5 ± 0.5	**0.004**
Foam, eye-opened, deg/s	2.2 ± 1.6	1.0 ± 0.9	**< 0.001**
Foam, eye-closed, deg/s	2.9 ± 1.1	1.9 ± 0.7	**< 0.001**
LOS			
Reaction time, s	0.9 ± 0.2	0.8 ± 0.1	**0.006**
Speed, deg/s	2.7 ± 0.9	3.4 ± 0.9	**< 0.001**
End-point excursion, %	54.6 ± 17.4	62.8 ± 10.2	**0.002**
Maximal excursion, %	71.7 ± 18.4	80.1 ± 9.7	**0.003**
Directional control, %	74.7 ± 11.0	79.8 ± 5.7	**0.002**
RWS			
Mediolateral sway, speed, deg/s	5.4 ± 1.2	6.0 ± 1.1	**0.004**
Mediolateral sway, directional control, %	76.9 ± 11.2	82.3 ± 4.6	**0.001**
Anterioposterior sway, speed, deg/s	3.2 ± 0.9	3.8 ± 0.5	**< 0.001**
Anterioposterior sway, directional control, %	64.7 ± 17.6	78.8 ± 8.6	**< 0.001**
Gait parameters			
Velocity, cm/s	40.3 ± 25.1	52.5 ± 20.5	**0.005**
Cadence, steps/min	90.7 ± 29.4	102.6 ± 17.4	**0.009**
Stride time, s	1.4 ± 0.6	1.2 ± 0.2	**0.027**
Stride length, cm	52.6 ± 30.0	64.4 ± 28.6	**0.035**
Step widths, cm	18.1 ± 2.8	16.4 ± 4.3	**0.020**
Swing phase, %^b^	28.0 ± 6.4	31.3 ± 3.2	**< 0.001**
Stance phase, %^b^	71.4 ± 7.1	68.7 ± 3.2	**0.007**
Double stance, %	43.8 ± 12.8	37.3 ± 6.5	**0.001**
Single limb support, %^b^	28.1 ± 6.4	31.3 ± 3.2	**0.001**

^a^Independent *t* test was used for age; otherwise, Mann–Whitney *U* test was applied for continuous variables. Chi-squared test was performed for sex; otherwise, Fisher’s exact test was applied for categorical variables.

^b^These parameters are calculated as the average of the left and right.

*JOA* Japanese Orthopedic Association, *mCTSIB* modified clinical test of sensory integration and balance, *LOS* limits of stability, *RWS* rhythmic weight shift.

### Ethical considerations and approval

All procedures were indicated and performed in compliance with our department’s standards and the Declaration of Helsinki and every participant of this study provided written informed consent. This study was approved by the institutional review boards at Kyung Hee University hospital (KHUH 2020-03-105-002).

## Results

A total of 62 patients with DCM were included (cervical spondylotic myelopathy 38 cases, OPLL 14 cases and herniated intervertebral disc 10 cases) and compared with 55 subjects without gait impairment as a control for clinical features of balance and gait (Table 2). No differences were found between two groups in age (*p* = 0.103) and sex (*p* = 0.778) Forty-one patients (66.1%) were male and mean age was 59 years (range, 27–82 years) in patients with DCM.

Among the 62 patients with DCM, the 10-step tandem gait grades were distributed as grade 0 (n = 1, 1.6%), grade 1 (n = 13, 21.0%), grade 2 (n = 14, 22.6%), grade 3 (n = 15, 24.2%), and grade 4 (n = 19, 30.7%), which was significantly differed from the results of control group (*p* < 0.001). The mean successful steps of tandem gait tests were significantly reduced as 7.1 ± 3.6 in patients with DCM compared to 9.9 ± 0.4 steps in patients without gait impairment (*p* < 0.001).

**Figure 3:**
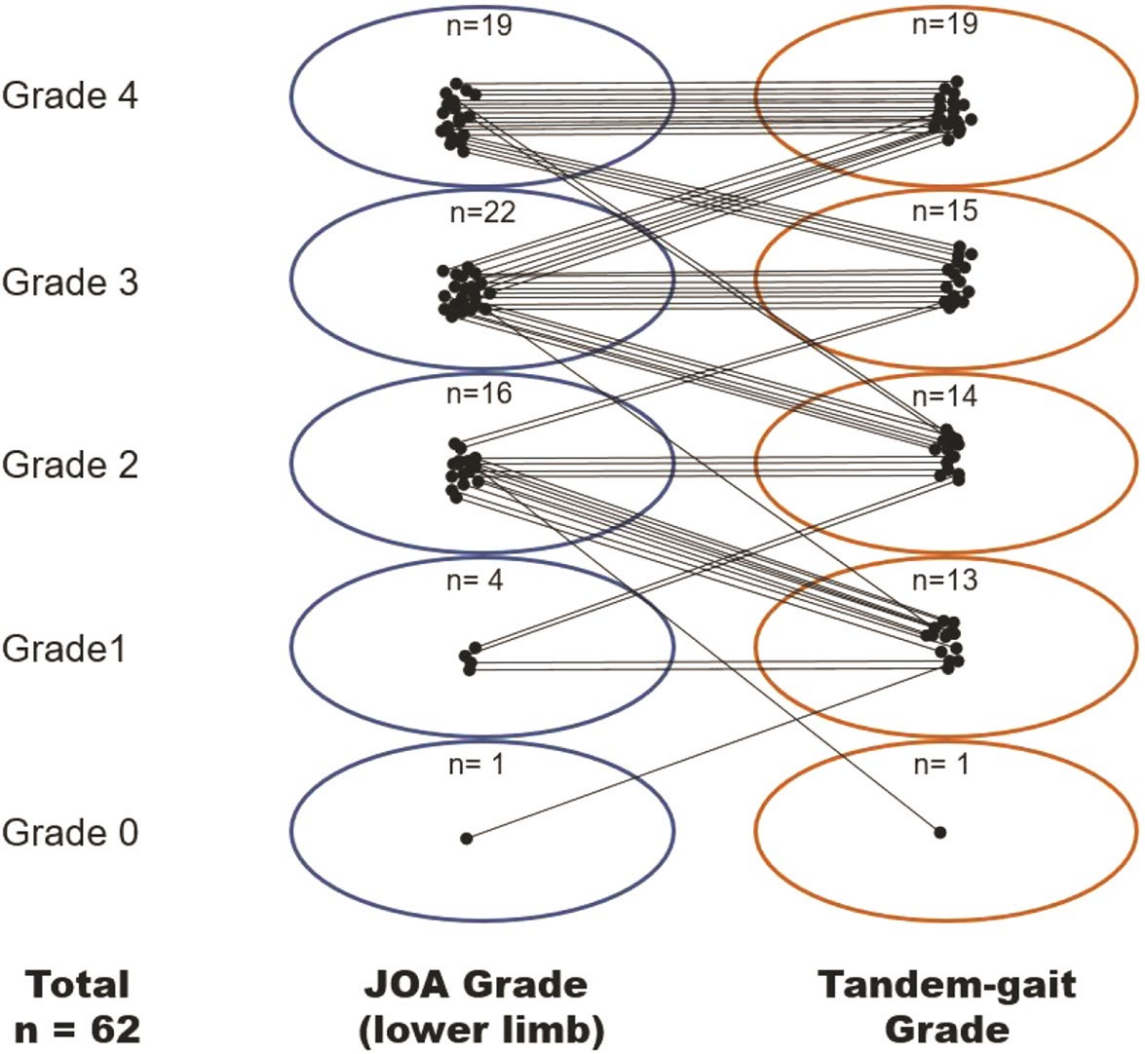
Each patient grade of the JOA sub-score (lower limb) and 10-step tandem gait test. Among them, 41.9% (n = 26) had same grades in both JOA sub-score and 10-step tandem gait test, but 38.7% (n = 24) and 19.4% (n = 12) showed higher grades in JOA sub-score and in 10-step tandem gait test, respectively.

The JOA subscores (lower limb) were distributed as grade 0 (*n* = 2, 3.3%), grade 1 (n = 2, 3.3%), grade 2 (n = 16, 26.2%), grade 3 (n = 22, 36.1%), and grade 4 (n = 19, 31.1%) in patients with DCM and none of the patients with Nurick scale 4–5 were included in the study. When matching the JOA subscores to the tandem gait grade for each patient (Fig. 3). 26 patients (41.9%) showed same grades in both JOA sub-score and 10-step tandem gait test, but 24 patients (38.7%) had higher grades in JOA sub-score than in tandem gait test and remained 12 patients (19.4%) showed lower grades in JOA sub-score than tandem gait grades.

The patients with DCM showed significantly different measures in balance and gait analysis compared to the subjects of control group (Table 2). Compared to the control, patients with DCM showed increased static instability in mCTSIB test and dynamic instability in LOS and RWS tests. Patients with DCM showed slow speed, reduced stride and cadence as well as increased stride time and step widths. In a gait cycle, swing phase and single limb support period were reduced in patients with DCM, while stance phase and double stance period were relatively increased compared to the control group.

The tandem gait grades were significantly correlated with the JOA subscore (r = 0.553, *p* < 0.001) and the Nurick scale (r = − 0.652, *p* < 0.001). The number of successful steps in tandem gait testing and tandem gait grades were correlated with quantitative spatiotemporal gait parameters including speed, stride time, stride length, swing phase/single limb support period, and stance/double stance phase (Table 3). Cadence and stride widths of gait parameters showed no correlations with tandem gait measures as well as JOA subscore and Nurick scale. In balance parameters, poor performance of tandem gait testing was correlated with severity of both static and dynamic instability in patients with DCM (Table 4). Speed of LOS test was associated with tandem gait measures, but not with JOA subscores and Nurick scales.

**Table 3:** Results of correlation analyses between gait parameters and tandem gait step/grade, JOA subscore and Nurick scale.

Variable	Tandem gait step (0–10)		Tandem gait grade (0–4)		JOA subscore (lower limb) (0–4)		Nurick scale (0–5)	
	rho	*p*-value	rho	*p*-value	rho	*p*-value	rho	*p*-value
Velocity, cm/s	0.612	< 0.001**	0.601	< 0.001**	0.621	< 0.001**	− 0.738	< 0.001**
Cadence, steps/min	0.253	0.053	0.238	0.069	0.203	0.117	− 0.104	0.448
Stride time, s	− 0.297	0.022*	− 0.255	0.051	− 0.340	0.007**	0.138	0.315
Stride length, cm	0.475	< 0.001**	0.479	< 0.001**	0.510	< 0.001**	− 0.678	< 0.001**
Step widths, cm	− 0.223	0.089	− 0.190	0.149	− 0.031	0.815	0.140	0.310
Swing phase, %	0.635	< 0.001**	0.620	< 0.001**	0.625	< 0.001**	− 0.647	< 0.001**
Stance phase, %	− 0.604	< 0.001**	− 0.579	< 0.001**	− 0.601	< 0.001**	0.618	< 0.001**
Double stance, %	− 0.629	< 0.001**	− 0.615	< 0.001**	− 0.664	< 0.001**	0.661	< 0.001**
Single limb support, %	0.625	< 0.001**	0.611	< 0.001**	0.662	< 0.001**	− 0.654	< 0.001**

*p < 0.05, **p < 0.01.

rho = Spearman’s correlation coefficient.

*JOA* Japanese Orthopedic Association.

**Table 4:** Correlation between balance parameters of static and dynamic stability and tandem gait steps/grade, JOA subscore and Nurick scale.

Category	Variable	Tandem gait steps (0–10)		Tandem gait grade (0–4)		JOA subscore (lower limb) (0–4)		Nurick scale (0–5)	
		Rho	*p*-value	rho	*p*-value	rho	*p*-value	rho	*p*-value
mCTSIB	Firm, eye-opened	− 0.510	< 0.001**	− 0.545	< 0.001**	− 0.346	0.006**	0.459	< 0.001**
	Firm, eye-closed	− 0.570	< 0.001**	− 0.639	< 0.001**	− 0.538	< 0.001**	0.471	< 0.001**
	Foam, eye-opened	− 0.589	< 0.001**	− 0.639	< 0.001**	− 0.629	< 0.001**	0.750	< 0.001**
	Foam, eye-closed	− 0.607	< 0.001**	− 0.680	< 0.001**	− 0.632	< 0.001**	0.718	< 0.001**
LOS	Reaction time, s	0.280	0.037*	0.208	0.124	0.157	0.239	− 0.394	0.004**
	Speed, deg/s	0.281	0.031*	0.262	0.045*	0.160	0.217	− 0.256	0.059
	End-point excursion, %	0.513	< 0.001**	0.486	< 0.001**	0.335	0.008**	− 0.559	< 0.001**
	Maximal excursion, %	0.525	< 0.001**	0.487	< 0.001**	0.340	0.007**	− 0.551	< 0.001**
	Directional control, %	0.608	< 0.001**	0.588	< 0.001**	0.410	0.001**	− 0.669	< 0.001**
RWS	Mediolateral sway								
	Speed, deg/s	0.441	< 0.001**	0.379	0.003**	0.327	0.010*	− 0.385	0.004**
	Directional control, %	0.592	< 0.001**	0.561	< 0.001**	0.501	< 0.001**	− 0.543	< 0.001**
	Anterioposterior sway								
	Speed, deg/s	0.466	< 0.001**	0.416	0.001**	0.386	0.002**	− 0.469	< 0.001**
	Directional control, %	0.591	< 0.001**	0.678	< 0.001**	0.524	< 0.001**	− 0.661	< 0.001**

*p < 0.05, **p < 0.01.

rho = Spearman’s correlation coefficient.

*JOA* Japanese Orthopedic Association, *mCTSIB* modified clinical test of sensory integration and balance, *LOS* limits of stability, *RWS* rhythmic weight shift.

## Discussion

At present, JOA score, modified JOA score, the neck disability index (NDI), Nurick scale grades, and Short-Form 36 are popular tools for evaluating patients with degenerative cervical diseases^16–18^. Among them, a subset of JOA score (motor function of the lower extremities) and Nurick scale have been most commonly used to reflect patient’s gait deterioration. However, when these tools are applied for assessing gait disturbance, accurate measurements are sometimes difficult because they depend on subjective questionnaires or decisions that are not easily distinguished between the grades. Until now, there has been no objective and appropriate evaluation method for the evaluation of gait dysfunction in patients with degenerative cervical diseases.

Tandem gait is considered a useful marker of dysfunction in neurologic conditions^19–21^. Since the early nineteenth century, tandem gait testing has been used for the neurological examination of lower extremity dysfunction in patients with cerebellar disease, parkinsonism, vestibulopathies, and other conditions. It is recognized as an integral part of the assessment of gait dysfunction, but there is no standardized or guideline-based protocol in actual clinical practice^10^. For quantitative evaluation, Morales-Bariceno et al.^22^ and Abdo et al.^23^ introduced the 10-step tandem gait test to differentiate patients with atypical parkinsonism from those with Parkinson’s disease. However, they used the 10-step tandem gait to assess only the presence of disease, but did not apply it to evaluate the degree of gait disturbance. So far, despite the long history and useful performance of the tandem gait test, not enough research has systematically evaluated gait disturbance using the tandem gait test. Moreover, few studies have used the tandem gait test to quantitatively assess gait dysfunction in patients with degenerative cervical myelopathy. Meanwhile, because the JOA and Nurick scales are subjective questionnaires, the severity of functional difficulty might be evaluated differently between individuals. However, this 10-step tandem gait test has the advantage of intuitive and clear-cut criteria between grades. The tandem gait test seems to be more objective and feasible than previous scoring system based on questionnaires, such as JOA and Nurick scale. If there was a space for 10-step tandem gait (about 3 m long flat floor), this test can be easily performed without subjective of the investigator.

In this study, the authors focused on whether there was a difference in the number of tandem gait steps taken depending on the degree of cervical myelopathy. The grades were divided (grades 0–4) according to the tandem gait step results and compared with previous tools for gait dysfunction assessment, such as JOA sub-score for lower extremities (scores 0–4) and Nurick scale (grades 0–5). In the results, the tandem gait step and grade showed significant correlations with gait parameters and the degree of association was comparable to those of the JOA sub-score and Nurick scale. The balance parameters showed higher correlation coefficients with the tandem gait grades and the Nurick scale grades than the JOA sub-scores. Because the 10-step tandem gait is easy to practice and the severity of the gait disabilities can be objectively differentiated between the grades, we believe that this 10-step tandem gait test would be an important screening test for the patients that have mild gait or balance problems, but feel like there is nothing wrong with their walking^24^. Furthermore, this test can be used for longitudinal monitoring for patients who have performed treatment for cervical myelopathy.

To create a standardized, guideline-based protocol using the tandem gait test, the authors determined the grades based on the number of tandem gait steps. A few previous studies determined whether or not there are gait abnormalities, based on the 10-step tandem gait test^22,23^. In this study, therefore, patients with a normal, 10-step tandem gait belonged to the highest grade (grade 4), whereas the patients who could not walk were in the lowest grade group (grade 0). During the evaluation of the cervical myelopathy patients, some patients could perform the 10-step tandem gait test but showed a slight left and right sway or unstable posture. These patients were considered to be different from the standard grade 4 criteria and were included in grade 3. Finally, based on the 3-step tandem gait test results, the patients who could do four or more tandem gait steps were included in grade 2 and the patients with three or fewer steps in the tandem gait test were grouped into grade 1. In general, patients with the first tandem gait step were often able to do two or three steps using walking recoil, but it was difficult to maintain four or more steps if gait abnormalities were evident. If it was possible to perform four or more tandem steps, patients would often make up to 10 steps. This is the reason why grades 1 and 2 were divided by 3-step tandem gait test results.

Clinically, although JOA scoring is universally used for evaluating various symptoms of cervical myelopathy due to its reproducibility and convenience, it takes not a little time depending the patient’s age or condition and sometimes it is quite difficult to distinguish between the grades due to its subjective questionnaires for both physicians and patients. Meanwhile, tandem gait test is world-widely used and considered as one of important neurologic examination for gait dysfunction. Because the tandem gait test is clearly visible and intuitive, it is easy for the patients to follow for the physicians to analyze. The authors directly compared the grades of JOA sub-score and 10-step tandem gait test and the results were presented in Fig. 3. In the results, 41.9% of patients had same grades in both JOA sub-score and tandem gait test, but 38.7% and 19.4% showed higher grades in JOA sub-score and tandem gait test, respectively. This difference is thought to be due to the subjective nature of the JOA sub-score. Actually, in our results (Table 4), tandem gait grade showed higher correlation coefficient with balance parameters than JOA sub-score and the patients’ grades in the tandem gait test appeared to be distributed, but the grades of JOA sub-score look to one side, including grade 2 and 3. Particularly, it is not easy to distinguish the exact differences between grades 1, 2 and 3 of JOA sub-score. Meanwhile, among the 19 patients of DCM patients with normal 10-step tandem gait, 7 patients had lower JOA sub-score (grade 3). Although this difference might be come from subjective characteristic of JOA sub-score grade 3rd query (including ‘slow’ walking), we thought that caution is needed in interpreting grade 3 of the 10-step tandem gait test.

There are some limitations of this study. First, there is a lack of results for test–retest reliability. Although we actually did test–retest of 10-step tandem gait for our subjects, the results of each 2 test were not shown in this study because only higher tandem gait score was collected. This can be one of important weaknesses. Instead, we recently made two times tandem gait tests of another patients group (n = 72) in out-patient clinic. Intra-observer reproducibility was high for two times tandem gait tests. Intraclass correlation coefficient of is 0.974. To be used universally, systematic evaluation for inter- and intra- observer reliability of the 10-step tandem gait test with a large scale will be necessary in future research. Second, the authors do not investigate association of the tandem gait measures with other variables, such as age, sex, height, leg length, shoe/foot size and so on. For a larger number of healthy subjects, these analyses are needed in order to fully understand the utility of novel outcome measure.

## Conclusion

Patients with DCM showed abnormal tandem gaits compared to the patients without gait impairment. The 10-step tandem gait grading system is correlated with symptom severity as well as quantitative balance and gait parameters, which suggests it could be used to evaluate balance and gait impairment in patients with DCM. The 10-step tandem gait is an objective and feasible screening test for evaluating gait impairment. The authors believe that this test would be a viable option for assessing gait disturbance in patients with DCM.

## Data Availability

All data analyzed during this study will be made available by the corresponding author on reasonable request.
